# What is resistance exercise? A review of current uses and potential ways forward

**DOI:** 10.1007/s00421-026-06148-2

**Published:** 2026-02-17

**Authors:** Lars H. Lohmann, José Afonso, David G. Behm, Stanislaw D. Siegel, Michael Keiner, Klaus Wirth, Anthony J. Blazevich, Eric R. Helms, Alyssa-Joy Spence, Konstantin Warneke

**Affiliations:** 1 Department of Human Movement Science and Exercise Physiology, University of Jena, Jena, Germany; 2 Centre for Research, Education, Innovation and Intervention in Sport (CIFI2D), Faculty of Sport, University of Porto, Porto, Portugal; 3 School of Human Kinetics and Recreation, Newfoundland and Labrador, Memorial University of Newfoundland, St. John’s, Canada; 4 German University of Health and Sport, Ismaning, Germany; 5 University of Applied Sciences Wiener Neustadt, Wiener Neustadt, Austria; 6 Centre for Precision Health, School of Medical and Health Sciences, Edith Cowan University, Joondalup, Australia; 7 Sport Performance Research Institute New Zealand (SPRINZ), Auckland University of Technology, Auckland, New Zealand; 8 Exercise Science and Health Promotion, Muscle Physiology Laboratory, Florida Atlantic University, Boca Raton, USA; 9 Chair of Sports Economics and Health Economics, University of Jena, Jena, Germany

**Keywords:** Resistance training, Effort, Intent, Intensity, Body weight, Plyometrics, Contraction time

## Abstract

The resistance exercise (RE) literature highlights the importance and impact of RE in sports performance, daily life, and clinical outcomes. While RE research dates back to the 19th century, current definitions of what constitutes RE are varied and give rise to questions about the requisites of RE. For example, some definitions refer to movements or muscle contractions against external resistance whereas others consider all repeated actions against one’s bodyweight. Taken to the limit, this could introduce confusion between RE and endurance exercise, for example. Without a clear definition, systematic reviews (with meta-analysis) discuss outcomes of studies examining the effects of RE in different settings, using heterogenous inclusion criteria (e.g., plyometrics may be included in some reviews but not in others). This may affect the direction and magnitude of effects, whereby it will be unclear if heterogeneous findings result from natural variation in response to RE or from different definitions of what constitutes RE. This challenges comparative analyses and may impair cohesive RE recommendations. Taken collectively, this obscures our understanding of RE effects. In an attempt to advance this understanding, the present review starts by mapping different types of definitions and illustrates their consequences. Secondly, this review provides a working definition of RE while discussing persisting challenges that require clarification before a consensus can be reached.

## Introduction

Resistance exercise (RE) research is an area of great interest within sports science. Despite its long history, established core concepts, and ubiquitous use, there is no universally accepted definition of RE and, as will be stipulated later, some definitions are mutually contradictory and others are simply too broad to be useful. Considering that the literature in general, and meta-analyses specifically, report positive RE effects on a vast range of outcomes irrespective of which training modalities are included under the umbrella of RE, one might argue the lack of an unambiguous definition is a minor problem. However, while the variance in effect sizes reported in meta-analyses might partly be explained by pooling effects from studies with heterogenous groups or stem from differences in training volume or intervention periods, variance likely originates from differences in the chosen intervention (i.e., training modality) as well.

This is problematic since systematic reviews with meta-analyses are considered the highest level of evidence (Murad et al. 2016; Wallace et al. 2022), are frequently used as the basis for general guidelines in many settings, and depend on method homogeneity. Consequently, these reviews should adhere to high standards as seen in the PRISMA 2020 guidelines (Page et al. 2021). In this context, PICO criteria connote *‘Participants*, *Intervention*,* Control*,* Outcome’* and highlight the importance of accurately classifying the interventions via in- and exclusion criteria to ensure the validity of the summarized evidence. Currently, systematic reviews on RE do not share a common definition of this *intervention* criteria (i.e., PICO) which leads to a potential scenario of ‘comparing apples to oranges’ (Fletcher 2007; Purgato and Adams 2012). This is exemplified by the fact that some RE reviews list plyometrics or calisthenics as inclusion (e.g., Steele et al. 2012; Lloyd et al. 2014; Pavlova et al. 2023; Mcleod et al. 2024; Nuzzo et al. 2024; Paluch et al. 2024) and others as exclusion criteria (e.g., Dankel et al. 2019; Wewege et al. 2022; Serafim et al. 2023). Changing definitions could severely affect the in- and exclusion criteria and with it the number of included studies. When excluding plyometrics, the Bragazzi et al. (2020) meta-analysis that investigated the effects of strength and plyometrics training on muscle strength under the umbrella of RE would need to remove more than one third of the previously included studies.

Albeit in adolescent populations, both Behm et al. (2017) and Zhu et al. (2025) determined very different magnitude effects on muscle strength outcomes with large effects for traditional strength (effect sizes of 0.88 and 1.49, respectively) and trivial effects for plyometrics (effect sizes of 0.16 and 0.09, respectively). Pooling effect sizes from plyometrics and traditional strength studies aiming to investigate RE effects on muscle strength outcomes might therefore yield results that are vague and imprecise because they would differ significantly when investigated separately. This heterogeneity threatens the validity and generalizability of the conclusions and ultimately impacts the academic understanding of RE efficacy and the development of evidence-based RE guidelines for practical application.

Consequently, this narrative review aimed to: (1) map definitional (in)consistencies by highlighting differences and recurring themes; (2) discuss the substance of these mapped criteria; and (3) propose a framework for a new RE definition while discussing why some included criteria need further refinement before a definitive consensus can be reached. Ultimately, this work intends to delve into the nuances of RE to critically highlight the limitations of current approaches while also synthesizing a considerable body of evidence to accelerate a future consensus paper.

## Definitional inconsistencies

The term RE entails two components: *resistance* and *exercise*. ‘Resistance’ hints that exercises covered by this term require the presence of some form of resistance (more on this later), while the ‘exercise’ component establishes the necessity for movement or at least activity. Herein lies the demarcation between RE and resistance training (RT) because training suggests the need for a systematic approach over time. Nonetheless, we consider the term RT in this review as well for two reasons: (1) RT differentiates itself from other training modalities through the ‘resistance’ aspect and (2) the term RT is extensively used throughout the literature and oftentimes synonymous with RE—indicating a relatively low discriminatory power between them. Still, in an attempt to maximize definitional accuracy, we argue that the addition of a criterion representing the ‘training’ component is substantial for RT.

A discussion around the ‘exercise’ part of RE would largely deviate from the goals of this review, so the focus will fall exclusively on the ’resistance’ component. We performed a narrative literature search on RE and RT definitions extracted from reviews (narrative, systematic, umbrella), position statements and stands, as well as guidelines. This investigation was initiated via a term-guided search in PubMed (date of search: December 15, 2025; search terms: “resistance exercise” or “resistance training”; sort by: best match). Since the aim was *not* to provide a systematic synthesis of definitions, the list of definitions in Table 1 is not exhaustive. The listed publications are examples of how the ‘resistance’ aspect is interpreted and exposes relevant inconsistencies and even contradictions. Additionally, we screened the definitions for additional aspects that may demarcate RE from other training modalities. Where possible, we added further information relating to the operationalization of the respective definitions.

**Table 1:** Common resistance training definitions and their implications

Author(s)	Article type	Topic	RT definition	Further information relating to RT definition or its application	Notable implications based on RT definition and further information
American College of Sports Medicine (1998)	Position stand	Cardiorespiratory and muscular fitness and flexibility	Not provided	“Resistance training should be […] of a sufficient intensity to enhance strength, muscular endurance, and maintain fat-free mass (FFM). Resistance training should be progressive in nature, individualized, and provide a stimulus to all the major muscle groups.” (p. 976)	Insinuates the necessity of an intensity threshold and progression for an activity to be considered RT.
Currier et al. (2023)	Systematic review with Bayesian network meta-analysis	Muscle strength and hypertrophy	“muscle contraction against external weight” (p. 1)	-	External weight requirement would exclude body weight-only modalities.
Grgic et al. (2021)	Narrative review	Skeletal muscle hypertrophy	Not provided	While the review does not define RT, the study clearly considers plyometrics to not be part of RT: “Objective: In this review, we critically evaluate studies directly comparing the effects of plyometric vs. resistance training on skeletal muscle hypertrophy.” (p. 530)	Would exclude plyometrics from RT.
Lloyd et al. (2014)	Position statement	Implementation in young populations	“Resistance training refers to a specialized method of conditioning whereby an individual is working against a wide range of resistive loads to enhance health, fitness and performance” (p. 1)	“Forms of resistance training include the use of body weight, weight machines, free weights (barbells and dumbbells), elastic bands and medicine balls.” (p. 1)	Resistance can be external or the person’s body weight.
Mcleod et al. (2024)	Umbrella review	Muscle strength, hypertrophy and physical function	“muscle contraction against external resistance” (p. 47)	“Resistance training programs (defined as the contraction of skeletal muscle against an external load/resistance, which can be provided by body mass, free-weights, guided motion machines, and elastic bands)” (p. 48)	Resistance can be external or the person’s body weight.
Nuzzo et al. (2024)	Narrative review	Muscle strength	“planned and repeated muscle actions against external resistance or one’s body weight” (p. 1140)	-	Resistance can be external or the person’s body weight.
Paluch et al. (2024)	Clinical statement and guideline	Cardiovascular disease	“exercise that evokes muscular contraction against an external force” (p. e217)	“RT can include free weights (ie, dumbbells), body weight (ie, push-ups, squats), machine weights, and resistance bands” (p. E222)	Body weight fulfils the external force criteria.
Pavlova et al. (2023)	Systematic review with meta-analysis	Tendinopathy management	“Exercise designed primarily to increase strength of muscles by causing them to produce substantive force against an applied resistance which can take several forms including the mass of the body or its segments, isoinertial resistance, elastic resistance, or strength training equipment such as isokinetic devices.” (Supplemental material SF2)	-	RE requires the expression of substantive force against a resistance that can be body weight.
Rodrigues et al. (2022)	Narrative review	Aging, sarcopenia and falls	“Resistance training is a form of periodic exercise whereby internal load or external weights provide progressive stimuli to the skeletal muscles to promote muscle mass and strength” (p. 5)	“Thus, resistance training includes moving limbs against resistance provided by own body weight, gravity, bands, weighted bars, or dumbbells.” (p. 5)	RT exercises needs to provide progressive stimuli. RT includes training against external weights but also works with body weight.
Serafim et al. (2023)	Systematic review	Exercise safety	“The American College of Sports Medicine (ACSM) defines resistance training for health and fitness as ‘a form of physical activity that is designed to improve muscle fitness by exercising a muscle or muscle group against external resistance’ [12].” (p. 2)	The authors state that “[r]esistance or strength training is widely performed in contemporary health and fitness environments through the use of equipment such as free weights, sectorized weight machines, plate loaded machines, weighted balls, resistance bands, and body weight resistance equipment [13]” (p. 2). Contradictory to this statement, on the same page, the PICOS framework in Table 1 lists—inter alia—calisthenics as an exclusion criterion for the intervention aspect. Also, the authors cite the 2009 ACSM position stand’s (American College of Sports Medicine 2009) RT definition, when in fact, the cited work did not define RT.	It is unclear whether body weight-based exercise fulfil the ‘external resistance’ criteria due to conflicting information within the manuscript.
Steele et al. (2012)	Narrative review	Cardiovascular fitness	“Resistance training appreciably covers a wide range of types of resistance, and it is acknowledged that in fact all movement occurs against resistance both internal and external” (p. 56)	“RT as an exercise modality was considered as exercise utilizing any of the following typical resistance types: free weights (including bodyweight exercise performed in the same characteristic manner), variable resistance machines, hydraulic resistance machines, and pneumatic resistance machines.” (p. 56)	Technically, all movements occur against resistance. Hence, body weight (e.g. walking) suffices as resistance. Is there a specific intensity, load, or volume necessary for RE? Specific listing of resistance types for machine-based training.
Steib et al. (2010)	Systematic review with meta-analysis	Muscle strength and function	“defined as an exercise where the subject exerts an effort against an external resistance or his or her own body weight” (p. 903)	-	Insinuates that a certain effort is necessary and that body weight fulfils the resistance criteria.
Wewege et al. (2022)	Systematic review with meta-analysis	Body fat percentage, fat mass, and visceral fat	“Resistance training interventions must have used a dynamic machine or free weight-based constant, external loads, and included at least one upper and one lower body exercise in the overall programme.” (p.288)	“We excluded studies that exclusively utilised bodyweight exercises and studies with concurrent nutritional interventions or additional exercise (e.g. aerobic exercise or team sport training).” (p. 288).	Body weight-only exercise and training against resistance bands would not qualify as RT.

*ACSM* American College of Sports Medicine, *ROM* range of motion, *RE* resistance exercise, *RT* resistance training

In essence, we identified two recurring themes that have the potential to inform the debate on how RE/RT demarcates itself from other modes of training, namely (1) source of resistance and (2) overload of the neuromuscular system. In the following, we will discuss the substance of each before proposing a new framework.

### Source of resistance and phenomenology

The question of what satisfies the *resistance* criterion is at the heart of the RE/RT debate. Hereby, the discussion around which implements are suited for RE introduces the demarcation dilemma.

#### External implements

Across publications discussing external implements (e.g., Steele et al. 2012; Lloyd et al. 2014; Paluch et al. 2024), there seems to be consensus that dumbbells, barbells, and dedicated RE machines fit the concept of RE. However, even for these traditional implements, a layer of complexity arises from small differences in terminology, namely ‘weight’ versus ‘resistance’—and whether these are synonyms. A vivid illustration can be found in recent reviews in high-impact journals defining RT as “muscle contraction against external weight” (Currier et al. 2023, p. 1) or “muscle contraction against external resistance” (Mcleod et al. 2024, p. 47). These were published in close temporal proximity on similar topics by virtually the same author group (first, second, and last authors are identical). While weight is characterized by its gravitational force, a resistance can be moved or lifted without exerting force against gravity, e.g., in pneumatic or hydraulic machines, while still including gravitational force through weight. This discrepancy is reflected in that some definitions listed in Table 1 specify the type of machine as “variable resistance machines, hydraulic resistance machines, and pneumatic resistance” (Steele et al. 2012, p. 56), “sectorized weight machines, plate loaded machines” (Serafim et al. 2023, p. 2), “machine weights” (Paluch et al. 2024, p. E222) or “weight machines” (Lloyd et al. 2014, p. 1). However, it is unclear if the authors planned on distinguishing between the source of resistance (e.g., weight versus pneumatic or hydraulic) within the group of machines. Similarly, it is unclear whether the differences in definitions by Currier et al. (2023) and Mcleod et al. (2024) (weight versus resistance, respectively) stem from considerations to differentiate between sources of resistance. Most likely, the listings for the machine types were not meant to be exhaustive and the terms weight and resistance were colloquially used as synonyms.

Apart from these traditional implements, further pieces of equipment receive attention in some of the definitions, namely medicine balls (Lloyd et al. 2014; Serafim et al. 2023), resistance bands (Lloyd et al. 2014; Rodrigues et al. 2022; Serafim et al. 2023; Mcleod et al. 2024; Paluch et al. 2024), iso-kinetic, and iso-internal devices (Pavlova et al. 2023). While resistance bands (band tension), iso-kinetic (pneumatic/hydraulic resistance) and iso-inertial devices (i.e., flywheels that enforce a moment of inertia (Hill 1920; Berg and Tesch 1994)) do not necessitate the presence of gravity, medicine ball training constitutes a classic case of actions against a fixed weight, and thus, a gravitational force. Further weighted implements that should be mentioned when discussing equipment—though not mentioned in the definitions in Table 1—are strongman implements such as stones, logs, kegs, bags and sleds (Keogh and Winwood 2017; Hindle et al. 2019).

Still, neither a listing of suitable implements nor a differentiation based on the terms weight or resistance are of substantive value. Firstly, there is no meaningful reason to exclude all sources of resistance except for gravitational force since the source of resistance seems to not matter in moderating the key RE/RT outcomes muscle size and strength (Balachandran et al. 2017; de Keijzer et al. 2022; Puustinen et al. 2023; Hu et al. 2024). Secondly, a list of equipment will almost inevitably neither be exhaustive nor meaningful in improving definitional accuracy because the way the implements are being used ultimately determines the training response. Consequently, we advocate against the inclusion of specific implements to an RE definition while proposing to favor the broader term ‘resistance’ over the narrower ‘weight’.

#### Body weight as a source of resistance

The debate surrounding gravity and implements leads to a similar discussion around whether bodyweight exercises that lack external weight/resistance satisfy the resistance criteria. This seems a matter of ongoing debate because some authors include bodyweight exercises in the definition (Steib et al. 2010; Steele et al. 2012; Rodrigues et al. 2022; Pavlova et al. 2023; Nuzzo et al. 2024) or indirectly through operationalization in the manuscript (Lloyd et al. 2014; Mcleod et al. 2024; Paluch et al. 2024). Others either do not mention them at all (American College of Sports Medicine 1998), exclude them (Wewege et al. 2022), or provide inconclusive or conflicting statements (Grgic et al. 2021; Currier et al. 2023; Serafim et al. 2023). While the review by Wewege et al. (2022, p. 288) “excluded studies that exclusively utilised bodyweight exercises”, Currier et al. (2023, p. 1) defined “muscle contraction against external weight”, and Grgic et al. (2021, p. 530) did not subsume plyometrics under the umbrella of RE as they “critically evaluate studies directly comparing the effects of plyometric vs. resistance training on skeletal muscle hypertrophy.” Finally, Serafim et al. (2023, p. 2) stated that “[r]esistance or strength training is widely performed […] through the use of equipment such as […] body weight resistance equipment” while also listing calisthenics as an exclusion criteria in their PICOS framework.

While it can be argued that ‘traditional’ RE and plyometrics exercises differ in their ability to enhance muscle strength (see the studies by Behm et al. (2017) and Zhu et al. (2025) in the Introduction), excluding plyometrics without providing a rationale to support such a decision is problematic because explosive accelerations of external objects (e.g., a heavy ball throw: $\:\mathrm{e}\mathrm{x}\mathrm{t}\mathrm{e}\mathrm{r}\mathrm{n}\mathrm{a}\mathrm{l}\:\mathrm{r}\mathrm{e}\mathrm{s}\mathrm{i}\mathrm{s}\mathrm{t}\mathrm{i}\mathrm{v}\mathrm{e}\:\mathrm{f}\mathrm{o}\mathrm{r}\mathrm{c}\mathrm{e}=\mathrm{m}\mathrm{a}\mathrm{s}\mathrm{s}\times\:\mathrm{a}\mathrm{c}\mathrm{c}\mathrm{e}\mathrm{l}\mathrm{e}\mathrm{r}\mathrm{a}\mathrm{t}\mathrm{i}\mathrm{o}\mathrm{n}$) would satisfy the lowest common RE definition denominator of ‘muscle contraction against external resistance’; similar to ‘loaded plyometrics’ (Rosas et al. 2016; Kobal et al. 2017). Disregarding the external resistance aspect and focusing on explosive bodyweight plyometrics performed without external loading, many plyometrics exercises still require high muscle forces, as often a large momentum in one direction must be decelerated and reaccelerated rapidly, usually with the load (the body) lifted upwards against gravity, e.g., in ballistic push-ups or jumps. This was already discussed in the 1970s by Norman and Komi who acknowledged that within a stretch-shortening cycle (SSC) “[…] a muscle is first stretched by an external force such as gravity, the inertia of a moving limb or by its own antagonist muscle” (1979, p. 245)_,_ meaning that the gravity acting on one’s body represents an external force. This reality is, inter alia, reflected upon by Mcleod et al. (2024) as well as Steele et al. (2012). While the review by Mcleod et al. defines RE as “muscle contraction against external resistance” (2024, p. 47), the intervention inclusion criteria (i.e., PICO) specify that the external load/resistance “can be provided by body mass” (2024, p. 48). Similarly, Steele et al. (2012, p. 56) elucidate that “in fact all movement occurs against resistance both internal and external”.

It must be questioned whether there is substantial value to discriminate RE from other exercise modalities solely based on the fact that a movement is externally loaded. This is especially relevant when factoring in that (a) 64% of one’s body weight must be moved during a regular and ~ 74% during a ~ 61-cm high feet-elevation push-up (Ebben et al. 2011) and (b) bodyweight push-ups and low-load bench presses produce similar adaptations in muscle strength and size (Kikuchi and Nakazato 2017). Additionally, bodyweight exercises are oftentimes more challenging for many who must, e.g., perform the lat pull-down exercise with a load smaller than body weight instead of performing pull-ups.

#### Phenomenology of exercises

Another aspect that might prove crucial to this discussion revolves around the outside view of movements. An argument can made for dynamic stretching to be considered RE as well. While there is no unique definition of dynamic stretching (Warneke et al. 2024b), it is generally described an action of controlled movement through the active range of motion (ROM) of a joint (Alizadeh et al. 2023) or as “controlled, dynamic movements in the end ROM” (Warneke et al. 2024b, p. 2). The works by Alizadeh et al. and Warneke et al. (2024b) both identify the similarities between RE and dynamic stretching: “one might propose that the actions [of RE] are similar to dynamic stretching albeit with an additional external load” (Alizadeh et al. 2023, pp. 712–713) and “eccentric contractions could also be defined as dynamic stretching” (Warneke et al. 2024b, p. 8). Taking this idea further, Kay et al. (2016) employed ‘active muscle stretching’ by means of a dynamometer stretching the plantar flexors through a predetermined ROM while subjects performed maximal eccentric contractions. This reasoning could explain why Alizadeh et al. (2023) included Pilates in their review on ROM effects following RT interventions.

Based on phenomenology alone, jumps could be considered an explosive squat and ballistic push-ups an explosive bench press. Both pairings appear similar, as they involve the same joint and muscle actions and utilize the SSC, i.e., an initial muscle lengthening that attenuates the force output during the following muscle shortening (Zatsiorsky et al. 2020). The literature differentiates fast (< 250 ms) from slow (> 250 ms) SSCs based on ground contact time (Schmidtbleicher 1992) with short SSCs found, e.g., in a drop and slow(er) SSCs in a countermovement jump. Hereby, it is important to note that incremental loading with external resistance results in longer transition times between eccentric and concentric muscle actions and thus longer SSCs (Suchomel et al. 2018). This can be seen for exercises that are commonly known as ‘loaded plyometrics’ (Rosas et al. 2016; Kobal et al. 2017). Upon sufficient increases in load, from an outside perspective, a countermovement jump loaded with a resistance that prevents an athlete from lifting fully off the ground resembles a traditional squat. Conversely, a heavily resisted squat (e.g., one-repetition maximum in the barbell back squat) could also be perceived as a plyometrics exercise because it uses a prolonged SSC and has explosive intent (i.e., maximal internal force production) (Behm et al. 2025). Likely, however, there is no hard-cut threshold but a gradated transition from plyometrics or dynamic stretching to RE and vice versa depending on contraction time and load. This would mean that a continuum exists in which, e.g., an exercise can start as ‘clearly’ plyometrics but through progressive loading may become full-on RE.

Consequently, another meaningful criterion of RE could be time under tension. In their bench press study on the effects of equalized time under tension on muscle strength and size, Martins-Costa et al. (2022, p. 1770) found that “training protocols with the same TUT [time under tension] promote similar strength gains and muscle hypertrophy” and “results indicate that training volumes cannot be considered separately from TUT [time under tension] when evaluating neuromuscular adaptations”. In line with that the latest meta-research on static stretching-induced muscle strength and size increases concluded the evidence insinuates that this training modality necessitates high volume (≥ 15 min per session) and frequent application (≥ 5 times per week) for each muscle to effectively moderate these parameters in humans (Warneke et al. 2024a). Traditional plyometrics, on the other hand, involve very little time under tension because of the relatively short ground contact times during which the muscles contract and work against resistance—and are still sometimes considered RE. Consequently, contraction time or time under tension might be a necessary but not sufficient RE criterion.

This is also in line with findings from Grgic et al. (2021) who determined that ‘traditional’ plyometrics exercise have the potential to induce significant muscle size increases, while also acknowledging that the body of evidence is limited to short-term interventions in previously untrained or recreationally active populations. Long-term interventions and studies in trained athletes are missing entirely; possibly because plyometrics cannot be sufficiently overloaded to foster long-term increases (Suchomel et al. 2018). This would fit the definition by Rodrigues et al. (2022, p. 5) who state RT should provide “progressive stimuli”.

### Long-term, progressive overload

Overload “is concerned with providing a proper stimulus for eliciting a desired physical, physiological, or performance adaptation” while “all stimuli will have a level of intensity, relative intensity (percentage of maximum), frequency, and duration (volume)” (Stone et al. 2000, p. 66). Of the included definitions, three (American College of Sports Medicine 1998; Steib et al. 2010; Pavlova et al. 2023) touch upon load (in terms of relative intensity) or magnitude of effort (i.e., intensity) while exercising; which might be a promising way forward. While Pavlova et al. (2023) state RT necessitates “substantive force against an applied resistance” in their Supplemental File 2, the definitions by the American College of Sports Medicine and Steib et al. directly mention intensity and effort, respectively, stating RT “should be […] of a sufficient intensity” (American College of Sports Medicine 1998, p. 976) and that it is exercise “where the subject exerts an effort” (Steib et al. 2010, p. 903).

#### Intensity

On a basic level, progressive RE necessitates progressive overload which “is the gradual increase of stress placed upon the body during exercise training” (American College of Sports Medicine 2009, p. 688). Hereby, the relative intensity appears to play a superordinate role since it is the most frequently employed criteria to delineate, e.g., endurance exercise from RT. Quantifying a baseline threshold, however, is problematic because the minimum intensity needed might be influenced through the interaction with other variables. Firstly, different volume-load combinations (e.g., low-load high-volume training) are potentially effective in increasing one of the main parameters of RT, namely muscle size (Schoenfeld et al. 2017; Lacio et al. 2021; Behm et al. 2023; Currier et al. 2023; Mcleod et al. 2024). Secondly, the relationship between volume and intensity will most likely differ based on the subject’s training level (Zatsiorsky et al. 2020).

Indeed, even regular walking (very high volume but very low relative intensity) demonstrated that a sole 6-month jogging intervention elicited an average ~ 9% muscle mass increase in untrained, older men (Schwartz et al. 1991). Effectively, running is “planned and repeated muscle actions against external resistance or one’s body weight” (definition by Nuzzo et al. (2024, p. 1140) that can significantly increase one of RT’s core outcomes. In a similar fashion, other very low intensity interventions such as swimming with hand paddles can be included as RE under definitions such as “working against a wide range of resistive loads” (Lloyd et al. 2014, p. 1). In contrast, Gamble (2006) found that for athletes who heavily depend on maximum strength in their respective sport, training load over the competitive season should not drop below 80% of the one-repetition maximum to prevent a decline in maximum strength. This vividly illustrates the loading disparity for typical RT outcomes based on the subjects’ training status.

We argue that a relative intensity criterion is crucial because leaving it out would severely reduce the discriminatory power towards endurance-oriented exercise. Still, the quantification of such criteria is difficult or impossible at this point because the evidence is vague and a matter of debate in the scientific community since most studies are short-term interventions and evidence on RT effects on muscle size in (highly) trained subjects is largely missing.

#### Effort

The scenario might be different for effort. While the definition by Steib et al. (2010) did not stipulate the magnitude of effort, in our opinion, growing evidence seems to indicate that a high magnitude of effort is necessary to enhance the key parameters muscle strength and size—at least long-term. The potential crucial importance of effort might also be inferred from the fact that different methods of RT or strength training are sometimes differentiated in their ability to create maximal muscular tension, namely maximal effort, repeated effort, and dynamic effort methods (Zatsiorsky et al. 2020).

Although the latest research insinuates that close proximity-to-failure (i.e., the level of exhaustion which relates to how far a subject is from the inability to perform further repetitions due to neuromuscular fatigue (Refalo et al. 2022)) seems less important for muscle strength improvements (Robinson et al. 2024), two major points support the idea that high effort is indeed needed for strength development. Congruent with the principle of specificity, the research shows a clear dependence of high-load RT when aiming to induce long-term maximal strength increases (Currier et al. 2023; Mcleod et al. 2024). When training with high loads (e.g., > 80% one-repetition maximum), this will, firstly, inevitably lead to (at least a relatively) close proximity-to-failure because of the inverse relation between load and volume and, secondly, demand a high degree of neural activation/drive to successfully complete the lift. Thus, high effort is still present. In the case of strength-focused RE, effort approximates the level and/or extent of the applied forces and the neural intent during force production. Indeed, when concentric velocity is performed without maximal intent, and is intentionally slowed, strength gains suffer (Hermes and Fry 2023).

The currently available literature for muscle hypertrophy seems more compelling in this regard indicating that high effort appears a potent stimulus for muscle size increases (Fisher et al. 2022; Refalo et al. 2023; Robinson et al. 2024); possibly of superior importance to overall training volume. Notably, Lasevicius et al. (2022, p. 346) determined significant quadriceps cross-sectional area increases within a 30% one-repetition maximum RT intervention only when sets were performed to momentary muscular failure and consequently “[…] conclude that when training with low loads, training with a high level of effort seems to have greater importance than total training volume in the accretion of muscle mass […]”. However, to date, no unambiguous threshold exists for any of the given effort measures which blurs the potential to determine a quantifiable effort measure.

For every rule there might be exceptions. For example, static stretching can, as mentioned in Sec. “Phenomenology of exercises”, promote muscle strength and size possibly without requiring high relative intensity or effort insofar these are coupled to voluntary action. A possible explanations might be that the ‘lack of relative intensity and/or effort’ is offset through the combination of high volume (≥ 15 min per muscle per session and ≥ 5 sessions per week) and high mechanical tension (stretching near the point of discomfort) (Warneke et al. 2024a). Additionally, two further aspects must be considered. Firstly, stretching to the point of discomfort (intervention studies by Warneke et al. (2022, 2023) state a 8/10 on a VAS scale while stretching for one hour daily) might be perceived as high relative intensity or effort, respectively. Secondly, the bulk of evidence stems from recreationally active subjects with only one available study in a trained population which is a n-of-1 trial in a highly-trained natural bodybuilder (Homer et al. 2025). As previously discussed, the subjects’ training level is an important consideration when appraising the interplay of different training variables that make up the training stimulus.

## Proposing a new framework with remaining gaps

Current RE and RT definitions emphasize phenomenology (e.g., is external resistance applied, what does the exercise look like from the outside) and further refining these aspects will most likely not yield meaningful results. Rather, an emphasis might be placed on other aspects that *can* advance the topic. Possibly, a meaningful delineation of RE/RT from other training modalities may lie in whether the exercise or training method allows the application of overload stimuli to the neuromuscular system which would enable *long-term* muscle strength and size increases. Consequently, we propose that a future RE definition encompasses criteria that relate to relative intensity, effort, and contraction time.

In our opinion, the term ‘muscle contraction’ covers the exercise part of RE sufficiently. Additionally, we advocate to specifically mention ‘internal and/or external’ in the context of resistance to not demarcate RE from other exercise modalities through unsubstantiated aspects such as source of resistance, implement, or exercise phenomenology.

While criteria referring to the two aspects resistance and exercise are indispensable for a comprehensive RE definition, we propose that the demarcation from other exercise modalities should rely on ‘internal’ aspects (e.g., how strenuous was the exercise on the neuromuscular system, was maximal intent applied, how high are the applied forces?) because these parameters ultimately drive long-term RE/RT adaptations. This will better reflect the practical training reality: when exercising with low effort and relative intensity, muscle strength and size will decrease long-term, irrespective of the exercise employed. Thus, even ‘traditional’ RE such as the barbell squat could fall outside the scope of RE.

Unfortunately, stipulating the magnitude or degree of intensity (i.e., effort) and relative intensity (i.e., load relative to the respective maximum) as well as quantifying a baseline time under tension is not straightforward. It also remains to be determined whether each would need to surpass a certain threshold or if there are ranges that depend on the interaction between them which in turn is also moderated by training status.

Based on available evidence, we propose the addition of ‘sufficient effort, relative intensity, and time under tension’ to acknowledge the facts that (a) most likely a sufficiently high magnitude of effort is necessary for long-term adaptations in muscle strength and size with RT that also relies on (b) sufficient time under tension to distinguish from ‘traditional’ plyometrics and (c) a baseline level of relative intensity because all-out endurance exercise can exhibit high degrees of effort and extended time under tension as well.

Perhaps the magnitudes/durations are continuums rather than fixed-point thresholds meaning there may be transitional ranges where, e.g., something would be endurance exercise, followed by a mixed endurance-resistance range and a RE part of the spectrum. This may not only apply to each aspect in its own right, but the interaction between them. Future research and consensus work is needed to increase the definitional accuracy of RE regarding effort, relative intensity, and contraction time and how their interactions moderate muscle strength and size outcomes in different populations.

For the time being and until new research emerges, we propose to add unquantified criteria with the intention of raising awareness to factor in these key aspects when engaging with RE. We propose a new RE definition that states: Resistance exercise is muscle contraction against internal and/or external resistance with sufficient effort, relative intensity, and time under tension given the current training status to induce long-term strength and/or muscle mass increases.

Generally, practitioners and researchers alike should critically question whether any given intervention actually provided an overload stimulus in the investigated population because this will differ vastly, e.g., between untrained populations and highly-trained athletes. This also applies to the magnitude of muscle strength gains and hypertrophy since small absolute and percentage increases could simultaneously prove substantial to elite-level athletes and negligible to untrained subjects. Researchers should additionally both (a) transparently state their methodological approach, especially towards effort and relative intensity, as well as (b) use more specific terminology in the classification of the training modalities to increase the informative value of their research.

## Data Availability

Not applicable.

## Abbreviations

RE: resistance exercise
ROM: range of motion
RT: resistance training
SSC: stretch-shortening cycle
